# Fixed Laws Governing Dental Amalgams

**Published:** 1916-02

**Authors:** N. K. Garhart


### ARE THERE ANY FIXED LAWS WHICH GOVERN THE BEHAVIOR OF DENTAL AMALGAMS?

BY N. K. GARHART.

There ought to be some fixed laws which govern the behavior of dental filling amalgams, even though they have not been recorded in past writings on dental metallurgy. There has been so much written upon this subject, and there has been such a wide divergence of opinion, that it would be of scientific interest to the profession if these fundamental laws could be isolated and recorded for future reference. After more than twenty-odd years of observation and experimentation I truthfully believe the following laws do exist:

### (1) Law of Insolubility.

The factor of insolubility increases in the same ratio as the proportion of silver is increased and the proportion of mercury is decreased in a given filling.

It has been fully determined by practical experience that all amalgams which have been solely made from base metals are soluble in the secretions of the mouth. The amalgam of copper and cadmium are examples. Now, insolubility begins as the noble metal silver is introduced into the formula, hence this factor increases as the percentage of silver is increased and the percentage of mercury is decreased in a given filling.

### (2) Law of Hardness.

The factor of hardness, or edge-strength, is increased in the same ratio as the percentage of silver is increased in a given formula until the "period of saturation" is reached, which is the point where the amalgam has reached its maximum degre of hardness. If the percentage of silver is carried beyond this point, then the degree of hardness begins to recede or grow less because the amalgam sets so rapidly that it is impossible to exclude the excess of this metal from the filling. I find this law to prevail as long as an excess of mercury is excluded from the filling.

### (3) Retention of Color.

Depends solely upon the factor of hardness and not to the presence of gold, platinum or zinc in the formula. A very hard and highly polished surface resists the sulphiding and oxidizing action of the saliva; also resists the abrasive action of mastication. Therefore, the "law of hardness" governs the subject of "color." As the degree of hardness is increased in a given formula the retention of color is increased in a like proportion.

(4) Law of "Period of Saturation."

This is the period when the metals have been correctly proportioned so that the alloy has reached a "balanced" state. Now, this law also pertains to the correct proportion of mercury that a "balanced" formula requires to prevent any present or subsequent changes in the dimensions of the mass, or when the amalgam can not be made to develop any traces of shrinkage or expansion.

(5) Law of "Time of Final Setting."

It is the volume of time which is required to bring a given amalgam to its final state of setting. It is the length of time which is measured from the time setting action begins until it ends. This law governs the volume of shrinkage and expansion which may be measured in a given formula. The shrinking factor is the greatest when the amalgam requires the longest time to set. The shrinking factor becomes less in volume as the period of setting is shortened, and which process can be carried to the point where expansion sets in. Thus "quick-setting" alloys, or "balanced" formulas, will set in from six to eight hours, while those of the old-style plastic working formulas will require from twenty-four to forty-eight hours before setting action fully ceases. Hence the shrinking factor of a given amalgam increases in a direct ratio as the setting process is retarded, and therefore said shrinking factor decreases in a direct ratio as the setting process is shortened.

A slow process of crystallization or setting produces a finer texture to the amalgam when viewed under a microscope. A rapid process of setting produces a course texture. Each individual crystal is thus enlarged, thereby causing the mass to increase in volume. The "period of final setting" may be either lengthened or shortened by the alloy maker according to the formula he selects to manufacture for his trade. On the other hand, the setting period may be either lengthened or shortened by the dentist according to the percentage of mercury which his fillings are made to retain. It is just as important to balance the formula of the filling as it is to balance the formula of the alloy if shrinkage and spheroiding are to be avoided. Should a balanced alloy reveal traces of shrinkage in the mouth, quantitative analysis will reveal that it was unbalanced by the dentist should a surplus of mercury be found in the filling. If the analysis reveals a surplus of tin, then the formula was not balanced by the alloy maker. Thus you will note that these four basic laws make the subject of dental filling alloys exceedingly simple and easily understood.

### WHAT INFLUENCE HAS EACH METAL UPON THE FORMULA?

It is quite apparent that the factors of "insolubility" and "hardness" are governed by the presence of the noble metal silver in the formula, but even then the factor of "proportion" is of great importance. With respect to all other virtues which every high grade alloy possesses, they may be attributed to a harmonious proportioning of the various metals which make up the complete formula rather than to any single element in said formula. I find it to be an error that gold, copper, zinc, platinum, etc., impart this or that useful property to any formula of dental filling alloys. These metals merely act as a partial substitute for silver, but only in the way of quick-setting agents; but then their presence in the formula accounts for a reduction in the percentage of tin, which we must further account for as a cause for quicker setting action. When our amalgam theorists refer to silver as an expanding element and tin as a contracting one, I claim they are only partly correct. It is the harmonious proportioning of the metals, or the correct percentage of composition, which is responsible for all of the desirable properties that are to be found in our high-grade alloys of commerce. The efficiency factor of every formula always depends upon the use of a maximum percentage of silver with a minimum quantity of tin, while the efficiency of the amalgam depends upon the use of a minimum percentage of mercury.

It may occur to you that better results would be secured by weighing the alloy and mercury. Unfortunately, the most efficient alloys require a large excess of mercury to form a plastic mix. Setting action would take place during the mixing operation, providing the exact amount of mercury is used such as the fillings should be made to retain. The excess of mercury produces a state of plasticity and greatly retards the setting action. Setting action begins just as soon as the excess of mercury is removed from the "mix."

Our high-grade alloys of commerce do not unite readily with mercury, and for this reason they should be thoroughly and energetically kneaded in the palm of the hand as soon as a plastic mix is obtained. You must produce a thorough "mix," or one in which every particle of alloy has been completely united with the mercury.

The "mix" should not be made until you are ready to condense it in the cavity. Do not express the mercury from it until you have divided it into a number of small portions. When you are ready to use each portion, then wafer it in the usual manner and immediately introduce it into the cavity. Setting action should not commence until after the alloy is condensed in the cavity. The unused portions of the plastic mix should not be wafered until you are ready to use each one of them. When the plastic amalgam begins to crumble, it should be thrown away. If you rework it, or attempt to make it plastic by the addition of more mercury, fatal results will follow should you use it for producing a filling.—*New Jersey Dental Journal.*

